# Association of low shunt burden from PDA and adverse outcomes in premature infants

**DOI:** 10.1038/s41372-025-02437-4

**Published:** 2025-09-20

**Authors:** Chase Brandt, Hatice Dilara Mat, Adrianne R. Bischoff, Patrick J. McNamara, Danielle R. Rios

**Affiliations:** 1 University of Iowa, Roy J. and Lucille A. Carver College of Medicine, Iowa City, IA, USA.; 2 Division of Neonatology, Department of Pediatrics, University of Iowa, Iowa City, IA, USA.; 3 Department of Internal Medicine, University of Iowa, Iowa City, IA, USA.

## Abstract

**OBJECTIVE:**

Compare the incidence of death or adverse respiratory outcome in patients with low shunt burden from PDA to those with no PDA and evaluate secondary outcomes associated with PDA between groups.

**STUDY DESIGN:**

Retrospective cohort study of all infants born <30 weeks gestation from 8/2018 to 5/2023 with TNE in the first postnatal week. Two groups: no PDA burden and low PDA burden. Primary outcome was composite of death or adverse respiratory outcome.

**RESULTS:**

112 infants [no PDA (*n* = 69), low PDA burden (*n* = 43)] with mean gestational age and birth weight 27 ± 2 weeks and 1006 ± 310 g, respectively, were included. Baseline demographics were comparable with no difference in primary outcome (*p* = 0.2).

**CONCLUSION:**

Prolonged exposure to low-volume PDA shunt was not associated with increased risk of death or abnormal respiratory outcome. Findings highlight the importance of redefining eligibility criteria for PDA trials, based on adjudication of shunt volume, to limit enrollment to patients with moderate- to high-volume shunts.

## INTRODUCTION

Patent ductus arteriosus (PDA) is a common condition in premature infants affecting approximately 1/3 of infants born <30 weeks gestational age (GA) and up to 60% of those born <28 weeks GA [1]. PDA in the setting of prematurity has been associated with increased mortality and major neonatal morbidity, including intraventricular hemorrhage (IVH), bronchopulmonary dysplasia (BPD), pulmonary hemorrhage, and necrotizing enterocolitis (NEC) [1-5]. Despite extensive literature on PDA and its association with adverse outcomes, there remains significant debate regarding its appropriate management. First, it has been estimated that greater than 80% of PDAs will close spontaneously within the first postnatal week [6]. Second, medically assisted PDA closure has not shown a reduction in adverse outcomes, and treatment is associated with risks such as intestinal perforation [6]. Thus far, evidence of treatment benefit from randomized controlled trials is limited, leading to widespread adoption of a more conservative approach.

To better predict which patients are at the greatest risk for untoward events, some experts now emphasize the need to evaluate overall shunt burden and hemodynamic significance, with evidence of improved outcomes using a targeted approach to treatment [7]. One of the criticisms of published trials is the limited adjudication of hemodynamic significance, leading to the enrollment of patients with increased risk of spontaneous closure or where shunt volume is low, thereby biasing towards adverse effects of treatment [1]. PDA diameter in isolation or indexed to weight is only weakly correlated with shunt volume [8, 9]. Combining PDA diameter with markers of left heart volume/pressure loading and end-organ perfusion may enhance the appraisal of hemodynamic significance [10]. To this end, several scores have been created that include clinical and/or echocardiography criteria to determine hemodynamic significance and need for treatment [11-13]. Integral to this approach is the identification of low-volume PDA shunts and refraining from treatment. There is, however, a paucity of data regarding the safety of prolonged exposure to a low-volume PDA shunt. The primary aim was to identify low-volume PDA shunts in a cohort of premature infants and compare their outcomes to those with no PDA exposure. Our hypothesis a priori was that patients with low PDA burden will not have higher incidence of the composite of death or adverse respiratory outcome compared to those with no PDA burden.

## METHODS

### Study design and population

**Fig. 1:**
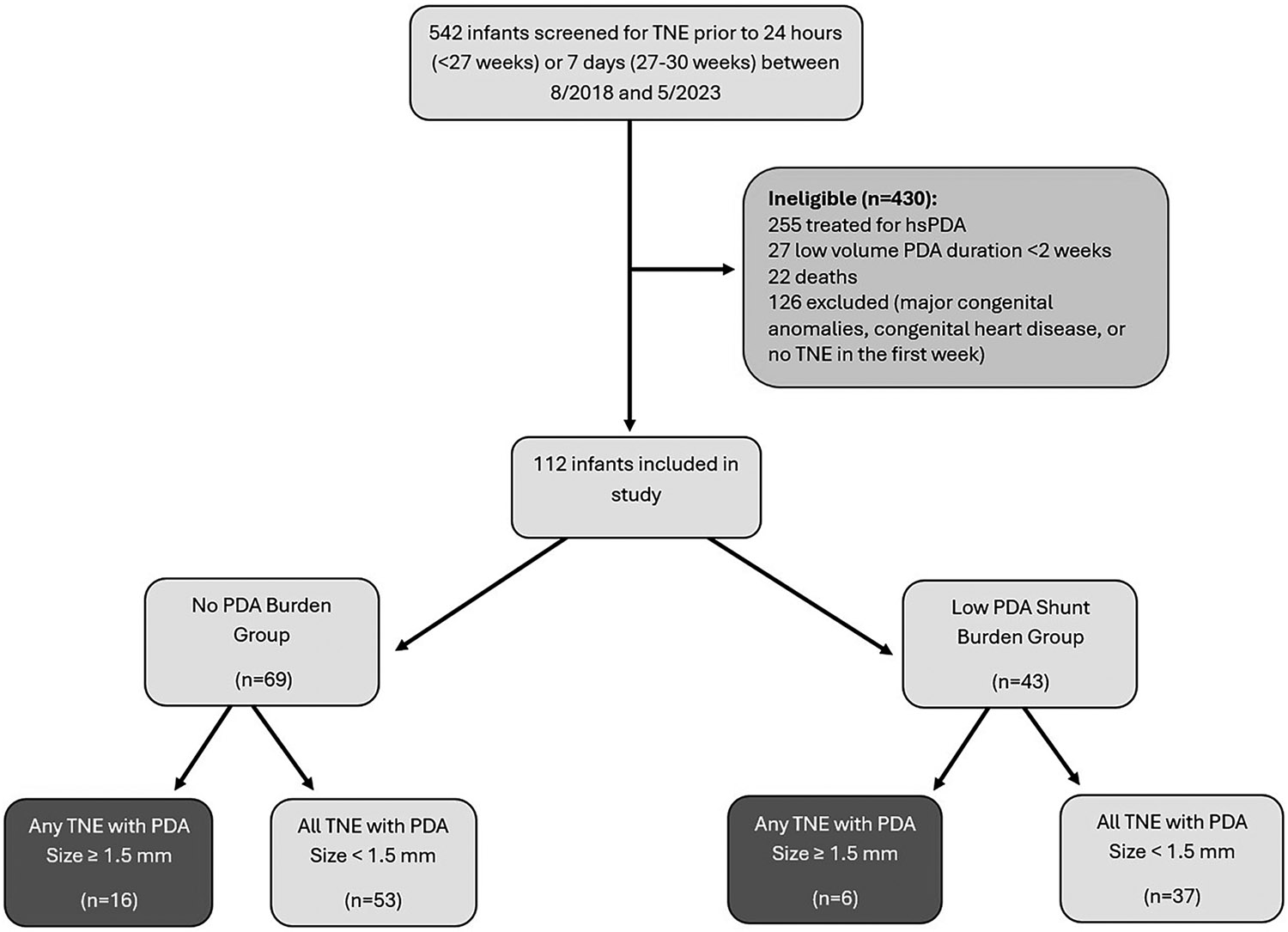
Patient flow and number with directly measured PDA ≥ 1.5 mm by Group. TNE targeted neonatal echocardiography, hsPDA hemodynamically significant patent ductus arteriosus, PDA patent ductus arteriosus.

This was a retrospective cohort study of premature infants born <30 weeks GA who were admitted to the Neonatal Intensive Care (NICU) at the University of Iowa Stead Family Children’s Hospital between August 2018 and May 2023. Neonates were selected from the admission and neonatal hemodynamics program databases. It is routine practice for all patients born <27 and 27–30 weeks GA to undergo screening targeted neonatal echocardiography (TNE) within the first 18–24 postnatal hours and first postnatal week, respectively. Patients with congenital anomalies, congenital heart disease (other than PDA, patent foramen ovale/atrial septal defect, or small (<1 mm) muscular ventricular septal defect), or where TNE was not performed within the first postnatal week were excluded (Fig. 1). The Iowa PDA score [11] (Table 1), is used to guide clinical management of the PDA.

### Ethics approval and consent to participate

The study was approved by the University of Iowa Institutional Review Board [ID 202203453] with a waiver of informed consent for all participants. All methods were performed in accordance with the relevant guidelines and regulations.

### TNE data collection

All infants underwent TNE within the first postnatal week using Vivid echocardiography system (GE Medical Systems Milwaukee, WI). All scans were performed and interpreted by experts in neonatal hemodynamics following a standardized protocol to ensure a comprehensive assessment of heart structure and hemodynamics [14]. Studies were stored in a dedicated archive for later research measurements and were analyzed on EchoPAC software (EchoPAC version BT10; GE Medical Systems) by a single trained operator blinded to the clinical course. The Iowa PDA Score, a multiparametric echocardiography scoring system, was utilized to determine the hemodynamic significance of PDA and to categorize patients into study groups [11]. This score serves as a surrogate marker for assessing shunt burden, evaluating left heart pressure, volume loading, and signs of systemic hypoperfusion. Additional indices of pulmonary blood flow and right and left ventricular function were also collected. Inter- and intra-observer variability were assessed on a minimum of 15 studies.

### Definition for groups

The No PDA burden group consisted of infants with spontaneous PDA closure either prior to the initial or on follow-up screening within the first postnatal week and no history of receipt of medical therapy for PDA during their hospitalization. The Low PDA Burden group consisted of infants with a PDA scoring <6 on initial screen within the first postnatal week, continued ductal patency with PDA score <6 for at least 2 weeks, and no history of receipt of medical therapy for PDA during their hospitalization.

### Demographic and clinical data collection

Patient demographics (e.g., GA, birth weight, sex, ethnicity), maternal history (e.g., age, obesity, smoking or substance use, gestational diabetes, chronic hypertension, preeclampsia, chorioamnionitis, antibiotic use, NSAIDS prior to delivery, antenatal steroids, history of intrauterine growth restriction), and delivery history (e.g., mode of delivery, resuscitation history, complications, APGAR scores) were collected from electronic medical records (EPIC Hyperspace). Parameters to assess cardiovascular and respiratory status at the time of TNE were evaluated, including oxygen saturation, systolic and diastolic blood pressure, mode of ventilation, fraction of inspired oxygen required (FiO_2_), respiratory severity score [calculated as mean airway pressure (Paw)FiO2∗], lactate, and cardiovascular medication therapy. Measures of neonatal morbidity during NICU admission, including length of stay, duration of intubation, and neonatal and respiratory outcomes that are known to be related to PDA and prematurity, were also collected.

### Blinding/data storage

Each patient was assigned an identification number (ID) in a master data file and study files only included ID numbers to maintain blinding to the clinical course. Echocardiography data were collected by a separate, trained expert blinded to the clinical trajectory and outcome data using a matched set of study identification numbers.

### Outcomes

Our primary composite outcome was the incidence of death prior to discharge or adverse respiratory outcomes between the groups. Adverse respiratory outcomes were defined as chronic pulmonary hypertension (CPH) prior to discharge and/or Grade 2 or 3 BPD. CPH was defined by TNE findings (RVSp ≥ 40 mmHg, eccentricity index ≥ 1.3, or paradoxical interventricular septal motion) on two studies separated by at least one week and/or receipt of pulmonary vasodilatory therapy (e.g., inhaled nitric oxide, sildenafil). Grade 2 or 3 BPD was defined according to the Jensen criteria [15]. Other neonatal outcomes included day 7 IVH classified according to Papile criteria [16], intestinal complications (NEC stage II or greater according to Bell’s criteria [17] or spontaneous intestinal perforation), severe retinopathy of prematurity (ROP) requiring Avastin or laser treatment, sepsis, pneumothorax, and systemic hypertension.

### Statistical analysis

Univariate analysis was performed to compare demographics, clinical characteristics, and echocardiography findings between the groups. Variables with a *p* value of <0.05 in univariate analysis were considered potential confounders and were further evaluated using logistic regression to assess their association with our primary outcome. In addition, GA was included in the model due to the known association with a higher risk of untoward outcomes. A *p* value of <0.05 was considered statistically significant. All statistical analyses were conducted using SPSS Version 28 statistical software [IBM, Armonk, NY, USA].

#### Sample size and power analysis.

A sample size calculation was performed to evaluate for a 15% change in the incidence of the primary outcome. We would have needed to study 166 case patients and 166 control patients to be able to reject the null hypothesis that the exposure rates for case and controls are equal with probability (power) 0.8. The Type I error probability associated with this test of this null hypothesis is 0.05. This study was powered to detect a 26% difference in the primary outcome, as it was not feasible to reach the sample size number required for a 15% difference. We used a sample of convenience, including all patients meeting eligibility criteria during the study period, since we would be unable to reach the necessary sample size in a reasonable time period.

## RESULTS

A total of 112 infants, whose mean GA and weight at birth were 27 ± 2 weeks and 1006 ± 310 g, respectively, were included. Of these, 69 infants were in the no PDA burden group and 43 were in the low PDA burden group with 12 and 5 being <25 weeks GA, respectively. Infants with no PDA were more likely to be inborn (*p* = 0.01); otherwise, baseline neonatal demographics and maternal characteristics were comparable between the groups (Table 2). There was no difference in the primary outcome of death or adverse respiratory outcome between the no versus low PDA burden groups [*n* = 33 (48%) vs *n* = 15 (35%), *p* = 0.2] (Table 3). There was no difference in the incidence of grade 3 BPD, CPH, pulmonary hemorrhage, duration of intubation, likelihood of being discharged on oxygen, or any other secondary outcomes analyzed between groups, except those with no PDA had a higher incidence of severe IVH (Table 3).

In total, 109 PDAs were identified, of which 25 (23%) measured ≥1.5 mm (Fig. 1) and 31 (28%) measured ≥1.5 mm when indexed to weight. Among the individual TNE markers of hemodynamic significance, infants in the low PDA burden group had higher mitral valve E wave (*p* < 0.001), mitral valve A wave (*p* < 0.001), and pulmonary vein S wave (*p* = 0.027) velocities (Table 4). In addition, infants in the low burden group had higher TAPSE (*p* = 0.048), RVs’ (*p* = 0.025), left ventricle output (*p* < 0.001), and right ventricle output (*p* = 0.04). Infants whose PDAs closed within the first postnatal week had larger PDA diameter (*p* = 0.003) and diameter indexed to weight (*p* < 0.001) as shown in Table 4.

Logistic regression showed that only increasing GA was associated with a reduction in our primary outcome (*p* < 0.001).

## DISCUSSION

In this cohort of infants born less than 30 weeks GA, we found that the rate of death or adverse respiratory outcome in patients with prolonged exposure to low-volume PDA shunt was comparable to patients whose PDA closed spontaneously within the first postnatal week. In addition, there were no differences in the incidence of death, BPD, CPH, or other secondary outcomes (any IVH, intestinal perforation, NEC, severe ROP requiring treatment, or systemic hypertension) between groups except those with no PDA had higher rates of severe IVH than those with low PDA burden. Regarding TNE measurements, several differences reached statistical significance, though they are unlikely to be clinically significant. Most measurements were slightly higher in the low burden group, with some achieving statistical significance. Among these, the only parameter suggestive of left heart pressure or volume loading was the mitral valve E wave velocity, which remained lower in both groups than the cutoff point to receive a point in the Iowa PDA score. Of note, we found those infants in the no PDA burden group and, therefore, with their PDA closed within the first postnatal week had PDAs that measured larger in diameter than those in the low burden group. In addition, we observed that a portion of patients in this cohort were born at less than 25 weeks GA and did not require treatment for PDA during their hospitalization. This further highlights the need to ensure accurate diagnosis of moderate to high-volume PDA shunt even in the youngest gestation infants. In summary, these data collectively support the hypothesis that, independently, ductal diameter is a poor surrogate of shunt volume and does not reliably predict the clinical course of infants with PDA; hence, clinical trials which enroll patients based on this measurement alone are questionable.

While this study is hypothesis generating, due to the inability to recruit the required number of patients for a properly powered analysis, our data still has direct relevance to the population of infants enrolled in clinical trials. As previously mentioned, randomized controlled trials to date have not demonstrated a clear treatment benefit for PDA. Central to the discussion of the validity of the findings of these trials is the adjudication of PDA as a true disease. Of note, Zonnenberg showed wide variance in establishing the diagnosis of PDA and/or how the PDA was categorized as hemodynamically significant [18]. A quarter of evaluated studies did not mention how PDA was diagnosed, and 10% did not include echocardiogram findings [18]. Many of the other studies used variable clinical and ultrasound findings. Many of the completed trials use arbitrary ductal diameter thresholds for enrollment, ranging from 1.5 to 2 mm [18], which limits the ability to distinguish between patients with low-volume versus moderate- to high-volume PDA shunts. For instance, the two recent trials, BeNeDuctus and Baby-OSCAR, which compared treatment with intravenous ibuprofen versus placebo, enrolled neonates with a ductal diameter of ≥1.5 mm without adjudication of shunt volume. It is noteworthy that neither trial found any difference in the incidence of death or BPD [19, 20]. The lack of diagnostic specificity may have contributed to the inconclusive outcomes of these studies by subjecting infants whose PDAs were more likely to close spontaneously to treatment. In our cohort, about 25% of infants had a ductal diameter (measured directly or indexed to weight) of 1.5 mm or larger, which may have qualified them for inclusion in previous randomized controlled trials. In addition, major biases in the estimation of transductal diameter using echocardiography vs. angiography (0.4 mm at the pulmonary end vs. 1.7 mm at the aortic end) may lead to misdiagnosis and inappropriate enrollment in PDA treatment trials [21]. These observations are noteworthy as our data suggests that some infants enrolled in clinical trials may have a low-volume shunt, which does not place them at increased risk for adverse outcomes. Not only do these data suggest these patients would not benefit from therapy for PDA closure, but exposure to treatment may also increase the risk of unintended side effects.

Medical therapy of PDA, while considered favorable to surgical ligation, is not without potential adverse effects. While the PDA is often pathologic in preterm infants, it may also be supportive (e.g., in the setting of pulmonary hypertension or undiagnosed congenital heart disease) or an inconsequential bystander [22]. Ibuprofen and indomethacin affect renal function, cause thrombocytopenia, and are associated with gastrointestinal bleeding [23]. Acetaminophen could contribute to increased pulmonary vascular resistance [24]. These are important observations as they suggest that the lack of treatment benefit and potential harm seen in previous trials, may relate to the exposure of patients without a hemodynamically significant PDA to unnecessary treatment. The use of TNE and a standardized scoring system to assess the hemodynamic significance of a PDA is, therefore, essential in this population. This approach ensures that only infants with a moderate- to high-volume PDA shunt, who have the greatest risk of adverse outcomes related to PDA, are selected for inclusion in future clinical trials. By refining the selection process, these trials can more accurately focus on those most likely to benefit from intervention.

## LIMITATIONS

This study has several limitations. First, these data are based on a small sample size and are representative of a single center, which affected the power and potential generalizability of the findings. It is plausible that there may yet be differences identified in a large sample size. Additionally, as a retrospective study, it relied on information documented in the electronic medical record, which could introduce variability in data quality. Furthermore, after the initial screening TNE, subsequent echocardiograms were performed at the discretion of the clinical team, leading to potential inconsistencies in the timing of follow-up assessments.

## CONCLUSIONS

In this retrospective cohort study, we showed that preterm infants with a prolonged exposure to a low-volume PDA shunt were no worse than those with no PDA exposure with respect to increased risk of death or adverse respiratory outcome. These findings highlight the importance of redefining eligibility criteria for PDA treatment trials based on comprehensive adjudication of shunt volume. Future trials should prioritize enrolling the infants with moderate- to high-volume shunts, as these are the infants most likely to experience adverse outcomes and, therefore, would most benefit from treatment while reducing risk for medication side effects.

## Figures and Tables

**Table 1.** Determination of PDA status on TNE, low-volume shunt score <6.

Iowa PDA Score (adapted from Rios et al)	0 points	1 point	2 points
Mitral Valve E wave velocity, cm/sec	<45	≥45 and ≤80	>80
IVRT, msec	>50	≥30 and ≤50	<30
Pulmonary vein D wave, cm/sec	<30	≥30 and ≤50	>50
Left atrium:Aorta	<1.3	≥1.3 and ≤2.2	>2.2
LVO:RVO	<1.5	≥1.5 and ≤2.5	>2.5
Diastolic flow reversal in descending aorta and/or celiac and/or middle cerebral artery	no	----	yes
PDA diameter[mm]÷weight[kg]	<1.5	≥1.5 and ≤3	>3

*PDA* patent ductus arteriosus, *IVRT* isovolumic relaxation time, *LVO* left ventricular output, *RVO* right ventricular output, *TNE* targeted neonatal echocardiography.

**Table 2.** Infant demographics and maternal characteristics: no pda versus low pda burden.

	No PDA Burden (*n* = 69)	Low PDA Burden (*n* = 43)	*p* value
Neonatal characteristics			
Birth weight, grams	971 ± 321	1064 ± 287	0.121
Gestational age, weeks	27.2 ± 2	27.4 ± 2	0.515
Gestational age <25 weeks	12 (17)	5 (12)	0.589
Male	32 (46)	20 (47)	1.000
Race			0.485
Black	10 (14)	7 (16)	
White	47 (68)	31 (72)	
More than one	6 (9)	0	
Other	6 (9)	5 (12)	
Hispanic	7 (10)	2 (5)	0.478
Intrauterine growth restriction	13 (19)	3 (7)	0.101
Type of delivery, vaginal	26 (38)	9 (21)	0.093
Delayed cord clamping	40 (58)	29 (67)	0.48
Multiple gestation	14 (20)	14 (33)	0.18
Breech delivery	1 (1)	1 (2)	0.723
Antenatal steroids	65 (94)	39 (91)	0.481
Inborn	64 (94)	32 (74)	**0.01**
Cord gas pH	7.26 ± 0.1	7.3 ± 0.1	0.084
Maternal characteristics			
Antenatal magnesium	54 (78)	29 (67)	0.978
Maternal antibiotics	62 (90)	32 (74)	0.244
Maternal smoker	14 (20)	8 (19)	0.892
Maternal SSRI use	16 (23)	7 (16)	0.626
Maternal preeclampsia	13 (19)	6 (14)	0.612
Preeclampsia with severe features	12 (17)	4 (9)	0.403
Chronic hypertension	4 (6)	1 (2)	0.653
Gestational diabetes	6 (9)	5 (12)	0.743
Maternal obesity	22 (32)	9 (21)	0.279
Chorioamnionitis	7 (10)	8 (19)	0.254

Data are presented in mean ± SD or frequency (%) as appropriate.

*PDA*, patent ductus arteriosus, *SSRI* selective serotonin reuptake inhibitor.

Bolded *p*-values are those that are statistically significant.

**Table 3.** Comparative analysis of adverse outcomes in neonates across groups.

	No PDA Burden (*n* = 69)	Low PDA Burden (*n* = 43)	*p* value
Primary outcome			
Death or adverse respiratory outcome	33 (48)	15 (35)	0.239
Respiratory outcomes			
Grade 3 bronchopulmonary dysplasia	2 (3)	1 (2)	1.000
Chronic pulmonary hypertension	4 (6)	0 (0)	0.296
Pneumothorax	2 (3)	0 (0)	0.523
Pulmonary hemorrhage	2 (3)	0 (0)	0.523
Duration of intubation (days)	17.5 [2, 38]	7 [3, 21]	0.152
Discharged on oxygen	34 (49)	19 (44)	0.559
Neonatal outcomes			
Death	0 (0)	0 (0)	1.000
IVH on day 7	13 (19)	4 (9)	0.278
Severe IVH (Grade 3 or 4)	9 (13)	0 (0)	**0.012**
NEC stage 2 or greater	3 (4)	1 (2)	1.000
Intestinal perforation	1 (1)	0 (0)	1.000
Severe ROP requiring avastin treatment	1 (1)	0 (0)	1.000
Systemic hypertension	3 (4)	0 (0)	0.284
Length of stay (days)	99.9 ± 61.8	82.6 ± 28.6	0.09

Data are presented as frequency (%), median [IQR], or mean ± SD.

*IVH* intraventricular hemorrhage, *NEC* necrotizing enterocolitis, *PDA* patent ductus arteriosus, *ROP* retinopathy of prematurity.

Bolded *p*-values are those that are statistically significant.

**Table 4.** Individual echocardiographic parameters: no PDA versus low PDA shunt burden group.

	No burden (*n* = 69)	Low burden (*n* = 43)	*p* value
Iowa PDA score components			
Mitral valve E wave velocity, cm/s	37.8 ± 8.8	43.4 ± 9.3	**<0.001**
IVRT, msec	49.9 ± 11.1	51.2 ± 11.2	0.448
Pulmonary vein D wave, cm/s	28.3 ± 8.5	29.1 ± 8.3	0.539
Left atrium: aorta	1.28 ± 0.2	1.33 ± 0.2	0.124
LVO:RVO	1.07 ± 0.5	1.07 ± 0.3	0.994
PDA size: weight, mm/kg	1.65 ± 1.1	1.06 ± 0.7	**<0.001**
Additional TNE information and markers			
PMA at the time of TNE	26.97 ± 2.1	27.33 ± 2.3	0.251
Weight at the time of TNE	0.85 ± 0.3	0.94 ± 0.3	0.066
Pulmonary vein S wave, cm/s	32.2 ± 8.3	34.9 ± 8.9	**0.027**
Mitral valve A wave velocity, cm/s	52 ± 9.6	59.2 ± 10.4	**<0.001**
Mitral valve E:A ratio	0.73 ± 0.15	0.74 ± 0.13	0.799
TAPSE, mm	5.2 ± 1.3	5.6 ± 1.3	**0.048**
RV S’, cm/s	4.9 ± 1	5.3 ± 1.1	**0.025**
RV FAC, %	0.46 ± 0.1	0.47 ± 0.1	0.492
EF by Simpson’s biplane, %	66.4 ± 7.2	67.7 ± 6.8	0.224
LVO, ml/min/kg	166.2 ± 42	192.6 ± 50	**<0.001**
RVO, ml/min/kg	171 ± 62	189.6 ± 58	**0.040**
PDA size, mm	1.36 ± 0.6	1.02 ± 0.5	**0.003**

Data are presented as mean ± SD.

*PDA* patent ductus arteriosus, *IVRT* isovolumic relaxation time, *LVO* left ventricular output, *RVO* right ventricular output, *TNE* targeted neonatal echocardiogram, *PMA* post menstrual age, *TAPSE* tricuspid annular plane systolic excursion, *RV* right ventricular, *FAC* fractional area change, *EF* ejection fraction.

Bolded *p*-values are those that are statistically significant.

## Data Availability

The data that support the findings of this study are available from the corresponding author, DRR, upon reasonable request.
